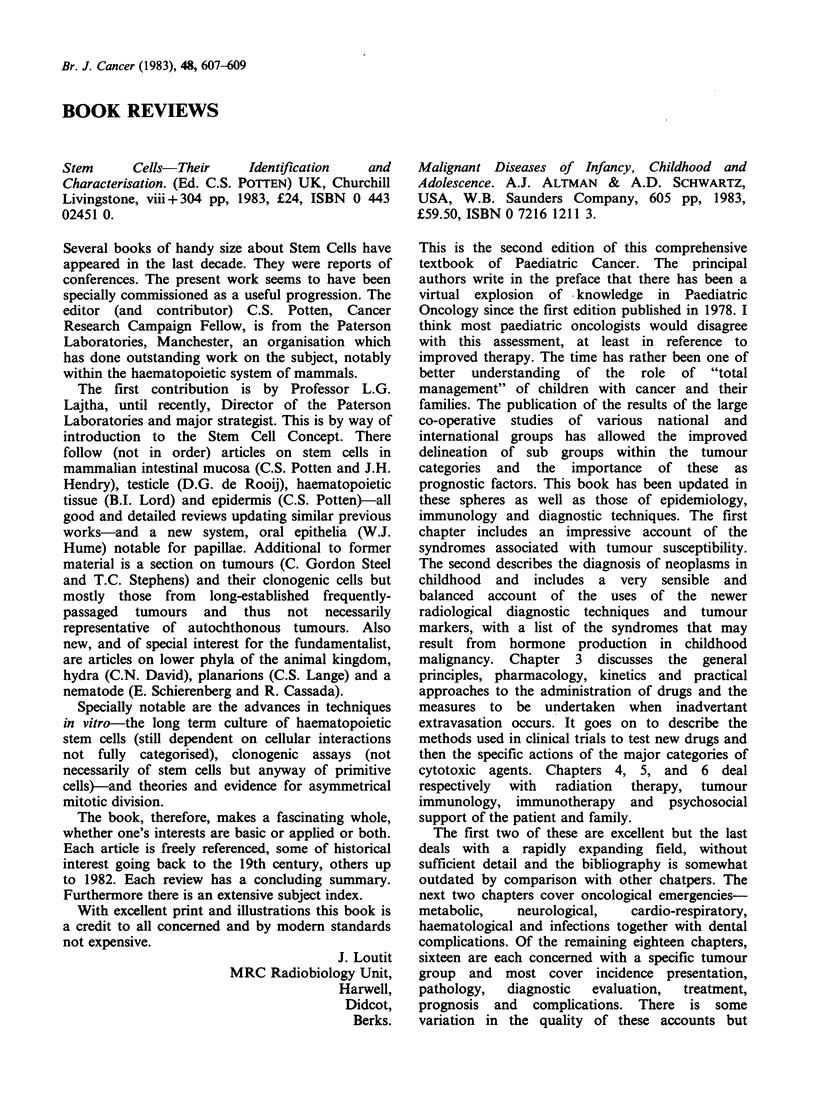# Stem Cells—Their Identification and Characterisation

**Published:** 1983-10

**Authors:** J. Loutit


Br. J. Cancer (1983), 48, 607–609

### BOOK REVIEWS

*Stem Cells—Their Identification and Characterisation.* (Ed. C.S. POTTEN) UK, Churchill Livingstone, viii+304 pp, 1983, £24, ISBN 0 443 02451 0.

Several books of handy size about Stem Cells have appeared in the last decade. They were reports of conferences. The present work seems to have been specially commissioned as a useful progression. The editor (and contributor) C.S. Potten, Cancer Research Campaign Fellow, is from the Paterson Laboratories, Manchester, an organisation which has done outstanding work on the subject, notably within the haematopoietic system of mammals.

The first contribution is by Professor L.G. Lajtha, until recently, Director of the Paterson Laboratories and major strategist. This is by way of introduction to the Stem Cell Concept. There follow (not in order) articles on stem cells in mammalian intestinal mucosa (C.S. Potten and J.H. Hendry), testicle (D.G. de Rooij), haematopoietic tissue (B.I. Lord) and epidermis (C.S. Potten)—all good and detailed reviews updating similar previous works—and a new system, oral epithelia (W.J. Hume) notable for papillae. Additional to former material is a section on tumours (C. Gordon Steel and T.C. Stephens) and their clonogenic cells but mostly those from long-established frequently-passaged tumours and thus not necessarily representative of autochthonous tumours. Also new, and of special interest for the fundamentalist, are articles on lower phyla of the animal kingdom, hydra (C.N. David), planarions (C.S. Lange) and a nematode (E. Schierenberg and R. Cassada).

Specially notable are the advances in techniques *in vitro*—the long term culture of haematopoietic stem cells (still dependent on cellular interactions not fully categorised), clonogenic assays (not necessarily of stem cells but anyway of primitive cells)—and theories and evidence for asymmetrical mitotic division.

The book, therefore, makes a fascinating whole, whether one's interests are basic or applied or both. Each article is freely referenced, some of historical interest going back to the 19th century, others up to 1982. Each review has a concluding summary. Furthermore there is an extensive subject index.

With excellent print and illustrations this book is a credit to all concerned and by modern standards not expensive.

J. Loutit
MRC Radiobiology Unit,
Harwell,
Didcot,
Berks.